# Prediction of Potential Suitable Habitats of *Cupressus duclouxiana* Under Climate Change Based on Biomod2 Ensemble Models

**DOI:** 10.3390/biology15020165

**Published:** 2026-01-16

**Authors:** Jialin Li, Yi Huang, Yunxi Pan, Cong Zhao, Yulian Yang, Jingtian Yang

**Affiliations:** 1 Forest Ecology and Conservation in the Upper Reaches of the Yangtze River Key Laboratory of Sichuan Province, Engineering Research Center for Forest and Grassland Disaster Prevention and Reduction at Mianyang Normal University of Sichuan Province, School of Life Sciences, College of Biology and Pharmacy, Mianyang Normal University, Mianyang 621000, China; lijialin@stu.mtc.edu.cn (J.L.);; 2 China College of Science, Tibet University, Lhasa 850012, China; hyhy1232021@163.com; 3 School of Environmental Science and Engineering, Southwest Jiaotong University, Chengdu 611756, China

**Keywords:** *Cupressus duclouxiana*, afforestation tree species, species distribution model, potential distribution, changes in distribution, three climate scenarios

## Abstract

*Cupressus duclouxiana* is an ecologically and economically important conifer endemic to southwestern China (e.g., central Yunnan and southern Sichuan), yet its potential distribution under future climate change remains insufficiently understood. In this study, we employed an ensemble species distribution modeling framework implemented in biomod2 to predict the current and future suitable habitats of *C. duclouxiana* across China. A total of 154 occurrence records and 17 key environmental variables were used to construct ensemble models integrating twelve algorithms. The ensemble model showed high predictive performance (TSS = 0.99, Kappa = 0.98). Temperature-related variables dominated habitat suitability, with the minimum temperature of the coldest month identified as the primary limiting factor, accounting for 44.1%. Under current climatic conditions, suitable habitats are mainly concentrated in southwestern China, particularly in Sichuan, Yunnan, and Xizang (Tibet). Future projections under three Shared Socioeconomic Pathways (SSP1-2.6, SSP3-7.0, SSP5-8.5) consistently indicate habitat expansion by the late 21st century, accompanied by pronounced northward and northwestward range shifts. The largest expansion is projected under the SSP3-7.0 scenario, highlighting the sensitivity of *C. duclouxiana* to intermediate warming trajectories. Overall, climate warming is expected to increase habitat availability while reshaping the spatial distribution of *C. duclouxiana* across China. These findings provide scientific support for climate-adaptive afforestation planning and conservation management, and offer broader insights into the responses of subtropical coniferous species to future climate change.

## Simple Summary

*Cupressus duclouxiana* is an important tree species for afforestation and ecological restoration in southwestern China, where it contributes to soil stabilization and regional forestry development. However, climate change may substantially alter the areas where this species can grow in the future. In this study, we used biomod2 ensemble species distribution models (SDMs) to predict the current and future suitable habitats of *C. duclouxiana* across China under different climate change scenarios. Our results indicate that winter temperature, especially the Minimum Temperature of Coldest Month, is the key factor limiting its distribution, while climate warming is expected to expand overall habitat availability. At present, the most suitable regions are mainly located in Sichuan, Yunnan, and Xizang (Tibet). In the future, suitable habitats are projected to shift northward and northwestward, with new potential areas emerging in Gansu and Inner Mongolia. These findings suggest that climate warming may create new opportunities for the cultivation of *C. duclouxiana* and highlight the need for climate-adaptive planning to support sustainable afforestation and ecological conservation.

## 1. Introduction

Climate change poses a serious challenge to natural systems in the 21st century. Observational records show a persistent increase in global mean surface temperatures over the past century, with accelerated warming in recent decades [1]. These changes have substantially affected ecosystems and biodiversity. In China, both the magnitude and rate of regional climate change exceed the global average over the same period [2]. Global warming has driven species to shift toward higher latitudes and elevations, increasing extinction risks [3], and has caused advances in spring phenology and delays in autumn phenology of plants [4], continuously reshaping their habitats and distribution patterns [5]. Therefore, investigating the potential suitable habitats of species is of great significance for plant introduction, cultivation, and management practices [6].

*Cupressus duclouxiana* (Cupressaceae, *Cupressus*) is native to the border region of Sichuan, Yunnan, and Guizhou provinces in southwestern China. With strong drought and cold tolerance and a well-developed root system, it is a key protective and afforestation species for soil stabilization and ecological conservation in mountainous areas of the region [7]. In the introduction area of Hanzhong, a five-year artificial introduction of *C. duclouxiana* has not significantly altered its natural leaf senescence rhythm, indicating that the phenological rhythm of *C. duclouxiana* is quite stable and well adapted to the climate of the introduction area. Compared with other conifer species in the same habitat, the phenological cycle of *C. duclouxiana* cone development is relatively longer [8], which may be one of the reasons for its higher seed set rate. This species exhibits robust ecological resilience, characterized by drought and cold tolerance, a straight trunk, and strong soil fixation capabilities. The decomposition of its litter significantly enhances soil water holding capacity and fertility, creating favorable conditions for the growth of understory companion species such as *Rosa roxburghii* and *Elaeagnus pungens* [9].

However, intensified global climate change, altered regional precipitation patterns, and anthropogenic disturbances have led to reduced growth rates, stand degradation, and frequent pest and disease outbreaks in coniferous forests, including *C. duclouxiana* [10]. Studies indicate that future climate change will cause significant migration or contraction of suitable habitats for common coniferous families such as Pinaceae, Cupressaceae [11], and Taxodiaceae [12], as well as broad-leaved families like Betulaceae and Fagaceae [13]. *C. duclouxiana* is relatively sensitive to temperature changes. For example, it is almost entirely not found in the northwest regions with large temperature differences, whereas in the central Yunnan Plateau and the eastern part of the Hengduan Mountains, where the terrain is high and valleys are deep with intense topographical fragmentation, major rivers such as the Jinsha River, Lancang River, and Yuan River form geographical barriers, influencing the distribution of the *C. duclouxiana* [8]. Currently, domestic research on *C. duclouxiana* focuses predominantly on its economic and ecological values. However, its potential distribution and dispersal dynamics under future climate change remain poorly explored. Addressing this gap is crucial for improving predictions of the geographic distribution of *C. duclouxiana* species and for supporting biodiversity conservation and sustainable resource management.

Single-algorithm SDMs have inherent limitations in predicting species ranges [14]. In contrast, the biomod2 ensemble modeling platform, based on the R programming language, employs a multi-model integration approach that yields more accurate and stable predictions than single models [15]. Biomod2 is a mature platform containing over ten common SDM algorithms [16], allowing users to freely combine models to maximize simulation precision and the accuracy of future distribution predictions [17]. Due to its ability to effectively improve prediction accuracy and reduce uncertainty, ensemble modeling has become a hotspot in species distribution research [18]. The biomod2 package encompasses ten mainstream algorithms: Generalized Linear Models (GLM), Classification and Regression Trees (CARTs), Gradient Boosting Machines (GBMs), Multivariate Adaptive Regression Splines (MARSs), Generalized Additive Models (GAMs), Artificial Neural Networks (ANNs), Flexible Discriminant Analysis (FDA), Surface Range Envelope (SRE), Maximum Entropy (MaxEnt) [19], and Random Forest (RF) [20]. In recent years, scholars have utilized the biomod2 package to integrate various single SDMs into ensemble models. This approach effectively compensates for the deficiencies of individual models and has been widely applied to simulate and predict the distribution patterns of protected plants, threatened species, and invasive species, yielding fruitful results [21,22,23].

This study integrates 154 valid occurrence records of *C. duclouxiana* with 17 key environmental variables screened for collinearity. The biomod2 ensemble framework combined with niche analysis was used to assess the potential geographic distribution, climatic suitability, centroid shifts, and niche dynamics of *C. duclouxiana* across China under current and future climate scenarios (SSP1-2.6, SSP3-7.0, and SSP5-8.5). Accordingly, this study aimed to (1) identify the key environmental factors shaping the distribution of *C. duclouxiana* and quantify their relative contributions; (2) characterize changes in potential suitable habitats and their migration directions under future climate change; and (3) assess niche stability and differentiation across climate scenarios to support cultivation zoning and the sustainable use of germplasm resources. Overall, this study contributes to climate-adaptive afforestation planning for *C. duclouxiana* in southwestern China and improves understanding of how subtropical conifers respond to climate warming.

## 2. Materials and Methods

### 2.1. Ecological Characteristics and Economic Value of C. duclouxiana

*Cupressus duclouxiana* Hickel is an evergreen conifer endemic to southwestern China, primarily occurring in southwestern Sichuan and northwestern to central Yunnan. The species typically inhabits regions characterized by a mild climate, dry winters and springs, and humid summers and autumns, and is mainly distributed in deeply incised valleys along the Jinsha River, Lancang River, and the western tributaries of the Yarlung Zangbo River.

**Figure 1:**
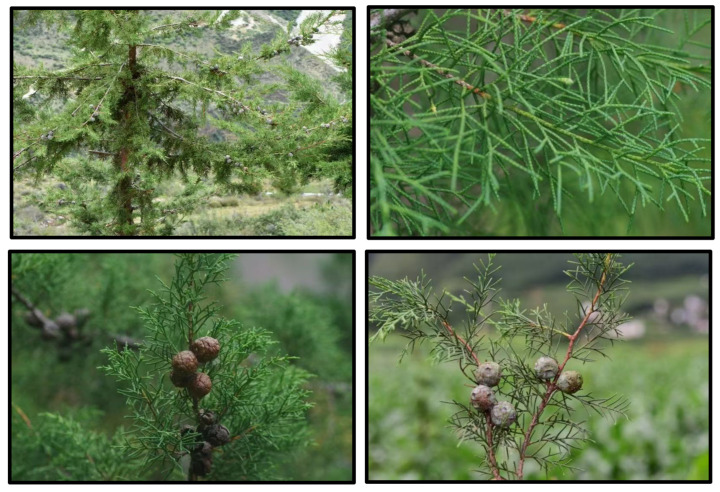
*Cupressus duclouxiana* in its natural habitat, photographed by Yang Jingtian and Jiang Hong.

The species is characterized by a straight trunk reaching up to 25 m in height and approximately 0.8 m in diameter at breast height, with grayish-brown bark and a broadly rounded crown formed by dense branching (Figure 1). Leaves are scale-like, oppositely arranged, about 1.5 mm long, bluish-green, and lack conspicuous resin glands, while the cones are globose, measuring 1.6–3.0 cm in diameter (Figure 1).

*Cupressus duclouxiana* exhibits high ornamental value and rapid growth in moist, deep soils, making it an important afforestation species in southwestern China with notable ecological benefits. Its wood is dense, fine-textured, durable, and aromatic, and is widely used in construction, furniture production, and carving, conferring considerable economic value.

### 2.2. Distribution Data Collection and Processing

**Figure 2:**
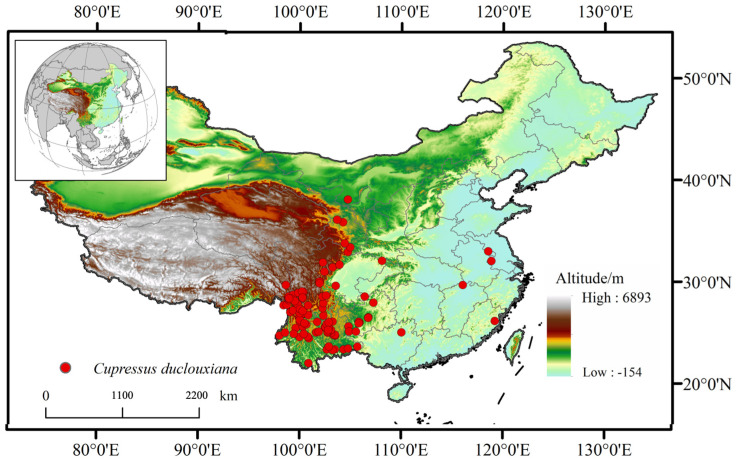
Distribution map of *Cupressus duclouxiana* in China.

In this study, occurrence records of *Cupressus duclouxiana* were compiled from multiple databases, including the Chinese Virtual Herbarium (CVH) [24], the Teaching Specimen Resource Sharing Platform of the National Science and Technology Resources [25], the Chinese Field Herbarium (CFH) [26], the National Specimen Information Infrastructure (NSII) [27], and the Plant Photo Bank of China (PPBC) [28], as well as from authoritative literature such as *Flora of China* and *Flora of Yunnan*. To reduce spatial sampling bias and avoid model overfitting caused by clustered records, duplicate and erroneous coordinates were removed using ArcGIS (version10.4.1) and the CoordinateCleaner (version 2.0.9) package in R (version 4.2.3). Spatial thinning was further performed using the EMTools (version 3.2.1) package at a resolution of 2.5 arc-minutes [29]. Additionally, a 5 km buffer was applied around each occurrence point, retaining only one record within each buffer to match the resolution of the environmental variables. After data filtering, a total of 154 valid occurrence records were retained for subsequent analyses (Figure 2).

### 2.3. Environmental Variables and Preprocessing

This study initially considered 29 environmental variables, comprising 19 bioclimatic factors, 5 soil properties, 3 topographic factors, one vegetation indicator, and one land-use and land-cover change (LUCC) variable. Current and future climate data were obtained from the WorldClim database [30]. Future climate projections were derived from the Beijing Climate Center Climate System Model (BCC-CSM2-MR). Compared with other CMIP6 models, BCC-CSM2-MR features higher atmospheric and land surface resolution, enabling a more detailed representation of terrain effects. As a result, it more accurately captures extreme temperature indices and their long-term trends across China and global land regions [31]. The model also performs well in simulating spatial variability in terrain-driven precipitation and local temperature, providing reliable climate inputs for species distribution modeling. In addition, relative to CMIP5, the Shared Socioeconomic Pathways (SSPs) represent an updated and more robust framework for simulating temperature and precipitation patterns and their trends, leading to improved climate projections at regional and global scales. This study incorporated three Shared Socioeconomic Pathways (SSP1-2.6, SSP3-7.0, and SSP5-8.5), representing increasing levels of greenhouse gas emissions. Soil and terrain data were derived from the Harmonized World Soil Database (HWSD) [32] developed by the International Institute for Applied Systems Analysis (IIASA). All environmental variables were resampled to a common spatial resolution of 2.5 arc-minutes (approximately 5 km at the equator) [33].

Before model construction, environmental predictors were filtered to minimize multicollinearity among climatic variables, which can adversely affect model performance. Initially, 19 bioclimatic variables were evaluated, along with species occurrence data, to assess their relative contributions to the distribution of *C. duclouxiana*. Pearson correlation analysis was then performed using ENMTools (version 3.2.1) to quantify relationships among the bioclimatic variables. For variable pairs with an absolute correlation coefficient ≥ 0.8, the predictor with the lower contribution was removed. The retained climatic variables were subsequently integrated with non-climatic factors, yielding a final set of 17 environmental predictors used for biomod2 modeling (Table 1).

### 2.4. Model Construction and Evaluation

Species distribution models were constructed using the biomod2 platform by integrating twelve single-algorithm SDMs (version 2.5). All algorithms were implemented with default parameter settings for initial model calibration and performance evaluation [34,35]. Model construction was performed using the BIOMOD_EnsembleModeling function, with 75% of occurrence records randomly selected for model training and the remaining 25% used for validation [36]. Each model was run ten times to improve robustness, and the prevalence parameter was fixed at 0.5 to balance species presence records and background points. Under this framework, a total of 240 single-model runs were generated [37].

To enhance predictive robustness and reduce uncertainty associated with individual models, an ensemble modeling approach was subsequently applied. Models with high performance were selected based on the true skill statistic (TSS), using a threshold of 0.8. Ensemble models were then constructed using three aggregation methods: mean, median, and weighted mean. The optimal ensemble strategy was determined according to overall evaluation performance and used for subsequent predictions of potential suitable habitats [38,39,40]. Model accuracy was assessed using three commonly adopted metrics, including TSS, the receiver operating characteristic (ROC) curve, and the Kappa coefficient, with evaluation criteria summarized in Table 2.

Ensemble projections were generated using the BIOMOD_EnsembleForecasting function. Continuous suitability predictions were transformed into binary presence–absence maps using the optimal TSS-based threshold [41]. Habitat suitability was then categorized into four classes: high (0.3 ≤ *p* ≤ 1.0), moderate (0.2 ≤ *p* < 0.3), low (0.1 ≤ *p* < 0.2), and unsuitable (0.0 ≤ *p* < 0.1). All model outputs were exported as raster (.tif) files for subsequent spatial analyses and visualization.

Finally, ArcGIS 10.8 was used to reclassify and visualize predicted suitable habitats under current conditions and future climate scenarios (2050s and 2090s; SSP1-2.6, SSP3-7.0, and SSP5-8.5), generating binary maps that illustrate spatial distribution patterns.

### 2.5. Centroid Migration

To analyze centroid migration patterns, the SDMTools package (v1.1–21) in R was used to calculate the centroid coordinates of suitable habitats for *C. duclouxiana* under different time periods and climate change scenarios. ArcGIS was then applied to extract the longitude and latitude of each centroid and to calculate migration distances between centroids. Finally, the resulting centroid shift matrices were imported into ArcGIS to visualize the spatial trajectories and directional trends of centroid migration for *C. duclouxiana*.

## 3. Results

### 3.1. Model Performance Evaluation

**Figure 3:**
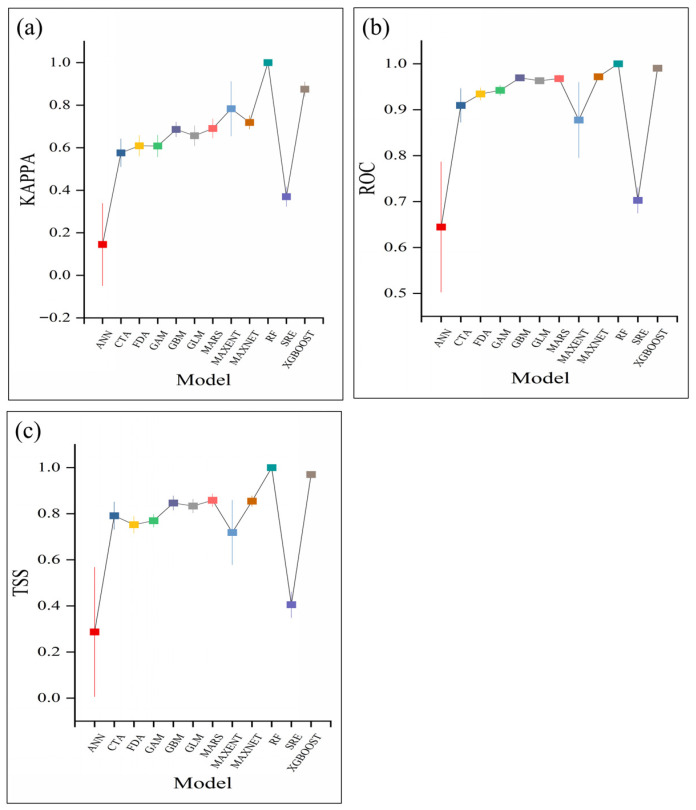
Evaluation of model accuracy using three metrics. (**a**) ROC; (**b**) KAPPA; (**c**) TSS.

All twelve models were successfully executed, generating a total of 120 model outputs (2 × 5 × 12). Model parameters were optimized using the BIOMOD_Tuning function [42], and model performance was evaluated during each iteration based on the selected metrics (ROC, KAPPA, or TSS). Evaluation scores were calculated for ANN, CTA, FDA, GAM, GBM, GLM, MARS, MAXENT, MAXNET, RF, SRE, and XGBOOST. Among all models, RF showed the best predictive performance for the potential spatial distribution of *C. duclouxiana*, with mean values of 1.00 for KAPPA, TSS, and ROC (Figure 3). XGBOOST ranked second, whereas ANN performed the poorest and failed to meet the accuracy threshold. Based on these results, the ensemble model achieved high predictive accuracy (KAPPA = 0.98, TSS = 0.99, ROC = 1.00), indicating strong overall performance and high reliability of the predicted results.

### 3.2. Environmental Variables Influencing the Potential Geographic Distribution of C. duclouxiana

**Figure 4:**
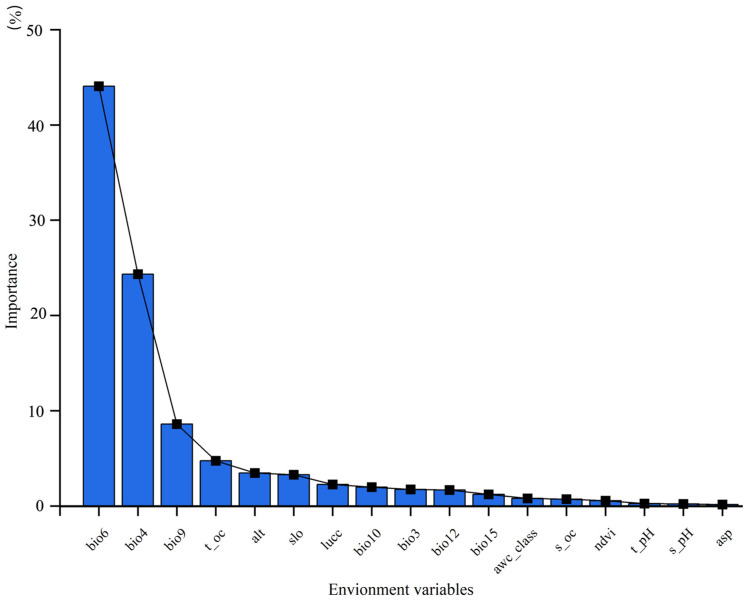
Relative importance of environmental variables in the ensemble model.

Environmental variables with relative contributions > 5% were considered dominant predictors. Among the 17 variables used for ensemble prediction, three exceeded this threshold: bio6 (44.1%), bio4 (24.3%), and bio9 (8.6%) (Figure 4). These results suggest that the potential distribution of *C. duclouxiana* is primarily controlled by temperature-related factors, particularly bio6 (minimum temperature of coldest month; 44.1%) and bio4 (temperature seasonality; 24.3%), indicating that thermal conditions exert stronger constraints than topography or soil properties.

**Figure 5:**
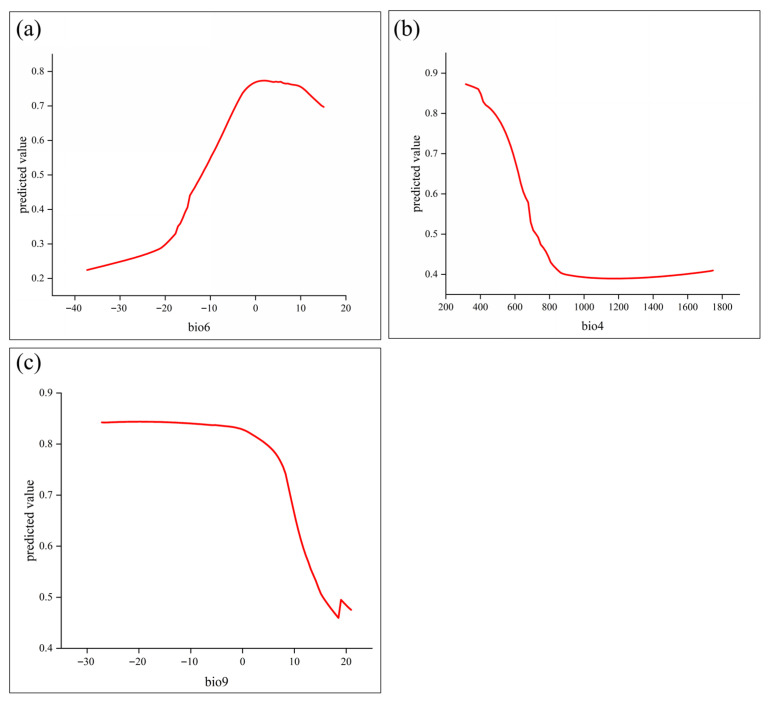
Response curves showing predicted occurrence probability of *Cupressus duclouxiana* along key environmental gradients. (**a**) bio6; (**b**) bio4; (**c**) bio9.

Response curves of ecological factors describe how habitat suitability varies with individual predictors, reflecting changes in modeled suitability along environmental gradients [43]. Because the environmental variable response curves of the ensemble model are not continuous and linear, this study employed the MaxEnt model (version 3.4.4) to generate continuous and linear response curves for environmental variables. To examine relationships between occurrence probability and key predictors, Figure 5 presents response curves for the three most influential variables: bio6 (minimum temperature of the coldest month), bio4 (temperature seasonality), and bio9 (mean temperature of the driest quarter). When predicted suitability exceeded 0.5, the corresponding environmental ranges were interpreted as optimal for species persistence. Specifically, suitable conditions were characterized by a minimum temperature of the coldest month above −10 °C, temperature seasonality (standard deviation) below 700, and a mean temperature of the driest quarter below 10 °C. Areas meeting these criteria were considered to represent the core suitable environmental space for *C. duclouxiana*.

### 3.3. Suitable Habitat Under the Current Climate

**Figure 6:**
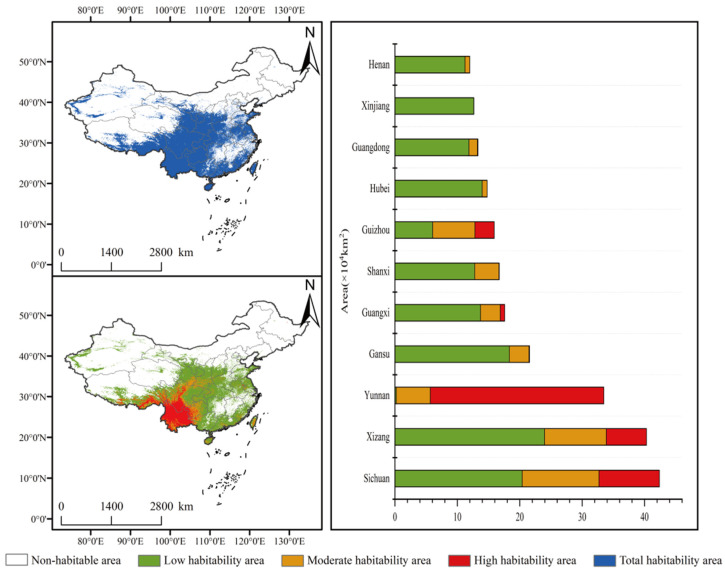
Predicted potential distribution of *Cupressus duclouxiana* under current climatic conditions and observed occurrence records.

Under current climatic conditions, suitable habitats for *C. duclouxiana* are mainly concentrated in southwestern and central China (Figure 6). The highly suitable area covers 4.83 × 10^5^ km^2^, accounting for 14.71% of the total suitable area in China. Highly suitable habitats are primarily distributed across southwestern China (Yunnan, Sichuan, and Xizang (Tibet)), with smaller patches occurring in Guizhou and Guangxi. Moderately suitable habitat covers 5.41 × 10^5^ km^2^ (16.48%) and expands outward from the highly suitable core, occurring mainly in Sichuan, Xizang (Tibet), and Guizhou. The low-suitability zone occupies the outermost layer of the suitability range, exhibiting a relatively dispersed distribution. Covering an area of 2.26 × 10^6^ km^2^ (68.81%), it is primarily located in Xizang (Tibet), Sichuan, and Gansu. Overall, habitat suitability for *C. duclouxiana* is highest in southwestern China, whereas suitability is relatively low in northeastern and northwestern China (Figure 6).

### 3.4. Projected Potential Distribution Under Future Climate Scenarios

**Figure 7:**
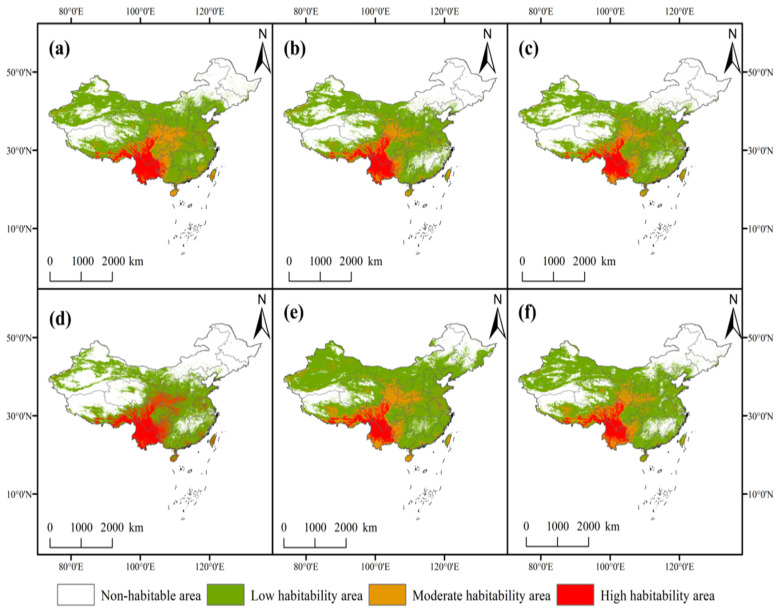
Potential geographic distribution of *C. Cupressus duclouxiana* during different future periods. (**a**) 2050s, SSP1-2.6; (**b**) 2090s, SSP1-2.6; (**c**) 2050s, SSP3-7.0; (**d**) 2090s, SSP3-7.0; (**e**) 2050s, SSP5-8.5; (**f**) 2090s, SSP5-8.5.

The projected potential distribution of *C. duclouxiana* under future climate scenarios is presented in Figure 7 and Table 3. Temporally, the total suitable habitat area generally exhibits an increasing trend from the 2050s to the 2090s.

Although the overall range of suitable habitats fluctuates significantly between the 2050s and 2090s, highly suitable habitats are projected to migrate northward in a diffusive manner, driven by future global warming and increased humidity. Under all three SSP scenarios, the total suitable area is projected to expand to varying degrees compared to the current distribution (Figure 7). Specifically, under the SSP1-2.6 scenario, the suitable area reaches 6.53 × 10^6^ km^2^ in the 2050s (an increase of 98.74% from the current area) but contracts to 4.75 × 10^6^ km^2^ by the 2090s (remaining 44.53% higher than current). Under the SSP3-7.0 scenario, the suitable area expands to 5.51 × 10^6^ km^2^ (a 67.80% increase) in the 2050s and further to 7.49 × 10^6^ km^2^ (a 127.91% increase) in the 2090s. Under the SSP5-8.5 scenario, the suitable area is 5.39 × 110^6^ km^2^ (a 64.37% increase) in the 2050s and reaches 6.16 × 10^6^ km^2^ (an 87.42% increase) in the 2090s.

Currently, the areas of high, moderate, and low suitability are 4.84 × 10^5^ km^2^, 5.61 × 10^5^ km^2^, and 2.26 × 10^6^ km^2^, respectively. By the 2050s, these categories are projected to increase by averages of 24.73%, 83.42%, and 85.83%, respectively. By the 2090s, the average increases are 15.22%, 97.40%, and 97.42%, respectively. The expansion is notably rapid in moderate and low suitability classes, indicating a transition from unsuitable to suitable habitats, as well as a shift from low to moderate suitability [44]. Overall, the total suitable area for *C. duclouxiana* is projected to increase by an average of 115.58% in the 2050s and 86.62% in the 2090s.

In summary, under future climate scenarios, the overall suitable habitat for *C. duclouxiana* shows a clear expansion trend compared to current conditions. By the 2090s, the suitable area is projected to increase by 44.53–127.91%. The species appears most sensitive to the SSP3-7.0 scenario, which yields the largest expansion magnitude (an increase of 127.91%), reaching a total area of approximately 7.49 × 10^6^ km^2^.

### 3.5. Overlap of Suitable Areas and Centroid Migration in Future Periods

**Figure 8:**
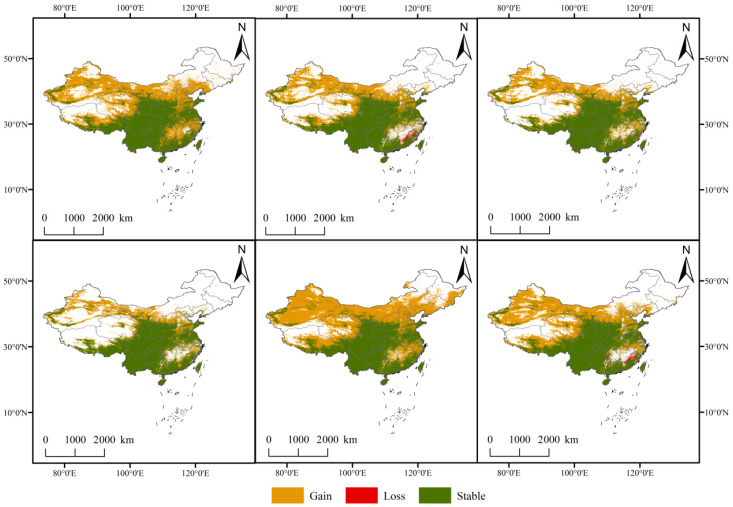
Spatial distribution pattern changes in *Cupressus duclouxiana* under different climate scenarios.

The overlap of suitable habitat areas in future periods is illustrated in Figure 8. By the 2090s, the vast majority of Yunnan Province is projected to remain suitable for the growth of *C. duclouxiana*. Under future climate scenarios, the distribution range of the species exhibits a continuous outward expansion associated with global warming (Table 4). This expansion pattern is observed both across increasing emission intensities within the same time period and over time within the same climate scenario. Geographically, the suitable area generally expands northward toward Gansu Province and the Inner Mongolia Autonomous Region, and eastward toward the northern parts of Shaanxi and Hebei Provinces, as well as the central region of Fujian Province.

**Figure 9:**
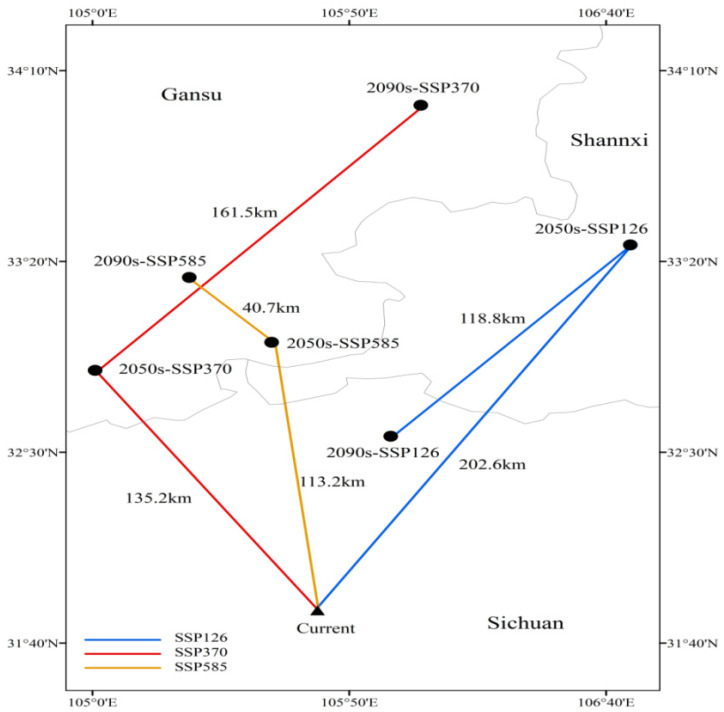
Centroid shifts in *Cupressus duclouxiana* under different climate scenarios.

Regarding centroid shifts (Figure 9), the distribution centroid of *C. duclouxiana* generally migrates northwestward under all future climate scenarios. By the 2090s, the centroid remains within Sichuan Province under the SSP1-2.6 scenario, whereas it shifts northwestward into Gansu Province under the SSP3-7.0 and SSP5-8.5 scenarios. The magnitude of centroid migration increases with the intensity of climate change.

## 4. Discussion

### 4.1. Model Predictive Performance

The predictive accuracy of SDMs is influenced by multiple factors, including sample size, the selection of environmental variables, and differences in modeling algorithms, which can introduce substantial uncertainty into model outputs [45]. Therefore, rigorous validation of model reliability is essential for obtaining credible predictions [46]. Accurate assessment of the impacts of climate change on species distributions is directly linked to effective species conservation, introduction, cultivation, and sustainable utilization strategies [47].

In this study, twelve different SDMs were implemented, and their predictive performance was evaluated prior to ensemble model construction. The results indicated that among the single algorithms, XGBoost and Random Forest (RF) exhibited the highest predictive accuracy. This is primarily because both methods are tree-based ensemble learning algorithms that effectively capture complex nonlinear relationships, reduce the risk of overfitting [48], and demonstrate strong robustness to noise and data imbalance, thereby often achieving high accuracy in species distribution prediction [49]. However, single models remain sensitive to algorithmic assumptions and parameter settings. For instance, although RF achieved the best performance in this study, it may still suffer from overfitting under certain conditions [41]. In contrast, ensemble models integrate the strengths of multiple algorithms and enhance robustness by offsetting individual model biases. Nevertheless, it should be noted that predictive accuracy also depends on model construction strategies, algorithm selection, and the quality of species occurrence data [50].

In addition, the predictive performance of SDMs generally declines with decreasing sample size [51]. Increasing sample size can reduce uncertainty in parameter estimation (e.g., mean values and predicted occurrence probabilities) [52], mitigate the influence of outliers [53], and better capture the complexity of species’ ecological niches across multidimensional environmental gradients. Larger datasets allow for more accurate characterization of species–environment relationships and their interactions [54]. It is worth noting that models performing well under large-sample conditions may not necessarily be suitable for small-sample scenarios [55], highlighting the need to further explore the trade-off between sample size and model complexity.

The ensemble model developed in this study achieved consistently high performance across multiple evaluation metrics, with TSS, ROC, and Kappa values reaching approximately 0.98–1.00. Its performance was at least comparable to that of the best-performing single models, while providing more stable and reliable predictions [9]. The slightly lower Kappa values relative to TSS and ROC are expected, as Kappa is more conservative due to its penalty for random agreement; nevertheless, a Kappa value of approximately 0.98 still indicates near-perfect agreement.

Based on the ensemble predictions, the highly suitable habitat area of *C. duclouxiana* was estimated to be 4.83 × 10^5^ km^2^, predominantly distributed in southwestern China, including Yunnan, Sichuan, and Xizang (Tibet), with smaller suitable areas occurring in Guizhou and Guangxi. By integrating specimen records from the Chinese Virtual Herbarium (CVH) [24] and distribution heat maps from iPlant [56], the observed occurrences of *C. duclouxiana* in Yunnan, Sichuan, and Xizang (Tibet) were found to fall largely within the predicted suitable regions. This concordance between empirical records and model predictions further supports the reliability of the distribution results for *C. duclouxiana*.

### 4.2. Environmental Drivers of the Geographic Distribution of C. duclouxiana

Climate is the decisive factor determining species distribution, and shifts in distribution ranges represent the most direct and evident indicators of climate change. At varying spatial scales, factors such as climate, historical distribution, topography, and soil properties significantly constrain species occurrences [57]. Our findings corroborate this principle: the minimum temperature of the coldest month (bio6), temperature seasonality (bio4), and mean temperature of the driest quarter (bio9) were identified as the dominant environmental drivers defining the suitable habitat of *C. duclouxiana*. The importance of these climatic variables far outweighed that of soil and topographic factors. Specifically, the contribution rate of the minimum temperature of coldest month (bio6) was 44.10%, indicating its critical role in shaping the habitat distribution. This finding is consistent with studies on species sharing similar niches, such as *Zelkova serrata* [2]. The potential reasons are as follows:

The minimum temperature of the coldest month is a critical climatic threshold governing tree survival, growth, and distribution. Its influence pervades physiological metabolism, morphological structure, and ecological adaptation strategies. Trees exhibit specific “physiological thresholds” for low-temperature tolerance; thus, bio6 directly determines whether a region can support the survival of a specific species. Even if temperatures do not reach lethal levels, low winter temperatures can still regulate annual growth efficiency by affecting physiological activities [58]. Previous studies using MaxEnt accurately predicted the potential distribution of *Zelkova serrata*, showing a similar dependence on the minimum temperature of the coldest month. These species exhibit low tolerance to extreme cold and drought, which limits their ability to expand into higher latitudes or more arid regions [59].

Regarding non-climatic variables, Topsoil Organic Carbon emerged as a key soil factor, reflecting the nutrient requirements for the slow growth of *C. duclouxiana* and its shallow root characteristics [60]. Among topographic factors, elevation emerged as an important predictor, indicating a preference for mountainous environments. Although soil and topographic variables contributed less than climatic factors overall, they may still play meaningful regulatory roles within specific microhabitats, particularly at finer spatial scales [61].

### 4.3. Relationship Between Habitat Area Changes and Environmental Factors Under Different Climate Scenarios

Climate change alters species ranges by modifying key environmental conditions, particularly temperature and precipitation, leading to shifts in geographic distributions [62]. By comparing three emission scenarios (SSP1-2.6, SSP3-7.0, and SSP5-8.5), this study demonstrates pronounced changes in the potential suitable habitats of *C. duclouxiana*.

Under the SSP1-2.6 scenario, the suitable habitat area expands rapidly during the early period and then increases at a slower pace. By the 2050s, the suitable area is projected to reach 2.50 × 10^6^ km^2^, an increase of 98.74% compared to the current distribution; by the 2090s, it reaches 4.74 × 10^6^ km^2^, an increase of 44.53% over the current area. This expansion is closely related to moderate temperature increases, optimized precipitation patterns, and the synergistic improvement of soil-topographic conditions. Rising temperatures alleviate thermal limitations in previously unsuitable regions (e.g., southern Gansu), while changes in precipitation further enhance water availability in these areas. Consistent with earlier research, climate warming has been shown to facilitate upward and poleward shifts in plant distributions [63]. These shifts frequently enhance suitability at distributional edges, particularly under low-emission conditions. In such scenarios, species with broad climatic tolerance tend to expand into marginal habitats [64], a response reflected in the expansion patterns identified here.

In contrast, under medium- and high-emission scenarios, suitable habitat shows sustained and rapid expansion throughout the projection period. Among these scenarios, SSP3-7.0 produces the most pronounced increase, with suitable area expanding to 5.49 × 10^6^ km^2^ (approximately 67.80%) by the 2050s and further to 7.46 × 10^6^ km^2^ (127.91%) by the 2090s. Under the more extreme SSP5-8.5 scenario, suitable habitat reaches 7.46 × 10^6^ km^2^ (about 64.37%) in the 2050s but declines to 6.14 × 10^6^ km^2^ (87.42%) by the 2090s. These patterns suggest that intensified greenhouse gas emissions enhance warming intensity and increase the frequency of extreme climatic events, particularly heatwaves and droughts, which can directly constrain habitat suitability for *C. duclouxiana*. High temperatures superimposed on drought stress can inhibit photosynthesis, disrupt water balance, lead to leaf chlorosis and abscission, cause growth stagnation, and in severe cases, induce dieback or mortality. Furthermore, such stress reduces the species’ resistance to pests and diseases [65]. Additionally, extreme climate events may indirectly alter the suitable habitat by affecting interspecific interactions.

### 4.4. Spatial Distribution Shifts in C. duclouxiana Under Climate Change

Under current climatic conditions, highly suitable areas for *C. duclouxiana* are primarily concentrated in southern Sichuan, western Chongqing, and northern/central Yunnan. Moderately suitable areas are distributed in southeastern Sichuan, southern Xizang (Tibet), and western Guizhou, while low suitability areas are found in central Xizang (Tibet), western Sichuan, and southern Gansu. This distribution pattern is highly congruent with the climatic characteristics of southwestern China, which are characterized by spring drought and summer–autumn rainfall, as well as by complex mountainous terrain [66].

Under future climate scenarios, rising temperatures and changes in precipitation drive a general northward expansion and spatial optimization of suitable habitats. By the 2050s, the total suitable area is expected to increase by an average of 76.97%, and by the 2090s, by 86.62% (Figure 7). Expansion regions are primarily concentrated in mid-to-high latitude provinces, such as Henan, Shandong, and Hebei. Concurrently, the ecological niche of *C. duclouxiana* shifts northwestward, whereas suitability in current core areas (e.g., central Yunnan and southern Sichuan) remains stable or increases slightly. Based on these shifts, future management strategies should prioritize the introduction and cultivation of high-suitability areas, including Dali, Lijiang, and Kunming in Yunnan, as well as Panzhihua and Xichang in Sichuan, to promote both ecological protection and economic benefits. Conversely, in ecologically fragile current habitats, such as western Sichuan, western Shaanxi, and eastern Guizhou, vegetation protection and stand modification should be strengthened to mitigate potential risks from extreme climate events. *C. duclouxiana* shows strong adaptability to future climate change, supporting its use in ecological afforestation and forestry development in southwestern China. The northwestward shift in its distribution centroid further indicates adaptive responses to climate warming [59].

Given the projected northwestward expansion of suitable habitats for *C. duclouxiana*, conservation and utilization strategies should integrate both current and future distribution patterns. In core distribution areas such as Sichuan and Yunnan, priority should be given to in situ conservation and long-term population monitoring to maintain genetic integrity. Concurrently, climate-adaptive introduction trials could be implemented in newly suitable regions, including southern Gansu and southwestern Inner Mongolia, to facilitate range expansion and enhance adaptive capacity. Sustainable utilization strategies should be based on multifunctional, close-to-nature forest management approaches that balance ecological protection with resource development. In addition, climate change risks should be explicitly incorporated into long-term management planning. Establishing a dynamic monitoring network, with particular attention to expansion frontiers and vulnerable marginal habitats, will be essential for tracking habitat changes and building a climate-resilient conservation and utilization framework for *C. duclouxiana*.

### 4.5. Limitations and Future Perspectives

This study employed a systematic modeling framework to predict the potential distribution of *C. duclouxiana* in China and to identify the key climatic factors constraining its distribution. It is worth noting that changes in the spatial scale of analysis (e.g., expansion or contraction of the study area) may lead to dynamic shifts in the thresholds of the identified environmental drivers [67]. In addition to climatic conditions, non-climatic variables such as vegetation cover can also exert significant influences on habitat suitability. However, due to the difficulty of reliably projecting long-term future vegetation dynamics, such variables were not incorporated into the current models. As a result, some areas predicted as potentially suitable may not be able to support viable populations in practice, highlighting the need to integrate localized information, such as hydrological and geological conditions, when applying model outputs at regional or site-specific scales [68].

Despite these limitations, the results of this study provide valuable guidance for large-scale planning and offer a solid foundation for the scientific management, cultivation zoning, and habitat conservation of *C. duclouxiana*. Global climate change reshapes ecosystems and indirectly yet profoundly influences the population structure and distribution dynamics of this species. At the same time, intensified human activities—including agricultural expansion, tourism infrastructure development, hydropower projects, and other industrial activities—have contributed to the rapid decline of wild populations [69].

To optimize afforestation planning for *C. duclouxiana* in southwestern China and to promote synergy between ecological protection and forestry development, future management strategies should be spatially guided by predicted suitable habitats while simultaneously incorporating multidimensional information, including topography, climate trajectories, ecosystem processes, population genetic backgrounds, patterns of human disturbance, and phylogeographic characteristics. Integrating these factors into comprehensive cultivation, management, and sustainable utilization schemes represents a central challenge for achieving the long-term conservation and rational use of this species. The analysis was limited to two projected periods (2050s and 2090s). Incorporating finer or continuous temporal resolutions in future studies may improve understanding of long-term habitat dynamics of *C. duclouxiana* under ongoing climate change.

## 5. Conclusions

This study systematically evaluated the impacts of climate change on the potential distribution of *C. duclouxiana* using an ensemble modeling approach. Temperature-related variables were identified as the dominant environmental drivers shaping the species’ potential geographic distribution under current climatic conditions. At present, highly suitable habitats are mainly concentrated in Sichuan, Yunnan, and Xizang (Tibet), while moderately and lowly suitable areas extend into surrounding provinces.

Under all three future climate scenarios, the total area of suitable habitat for *C. duclouxiana* is projected to expand. The centroid of suitable habitats is predicted to shift predominantly toward the northwest (Gansu and Inner Mongolia) and northeast (Shaanxi and Hebei), with greater climate change intensity associated with longer northwestward migration distances. These findings provide a scientific basis for afforestation zoning, conservation planning, and the sustainable utilization of *C. duclouxiana* under ongoing climate change.

## Figures and Tables

**Table 1:** List of environmental variables used for modeling.

Environment Variable	Abbreviation	Unit	Contribution Rate
Minimum Temperature of Coldest Month	bio6	°C	44.06%
Temperature Seasonality	bio4	°C	24.33%
Mean Temperature of Driest Quarter	bio9	°C	8.58%
Mean Temperature of Warmest Quarter	bio10	°C	1.98%
Isothermality	bio3	%	1.75%
Land-Use and Land-Cover Change	lucc	/	2.25%
Annual Precipitation	bio12	mm	1.67%
Precipitation Seasonality	bio15	%	1.21%
Elevation	alt	m	3.46%
Slope	slo	%	3.28%
Aspect	asp	◦	0.15%
Available Water Capacity	awc_class	%	0.79%
Topsoil Organic Carbon	t_oc	weight	4.75%
Topsoil pH	t_pH	−log(H^+^)	0.26%
Subsoil Organic Carbon	s_oc	%	0.72%
Subsoil pH	s_pH	/	0.22%
Normalized Difference Vegetation Index	ndvi	/	0.55%

**Table 2:** Evaluation criteria for model performance based on TSS, ROC, and Kappa.

Evaluation Metric	Poor	Fair	Moderate	Good	Excellent
ROC	0.50–0.60	0.60–0.70	0.70–0.80	0.80–0.90	0.90–1.00
TSS	0.00–0.40	0.40–0.55	0.55–0.70	0.70–0.85	0.85–1.00
Kappa	0.00–0.40	0.40–0.55	0.55–0.70	0.70–0.85	0.85–1.00

**Table 3:** Predicted areas of suitable habitat for *C. duclouxiana* under different future climate scenarios.

Climate Scenarios	Low Suitability Area		Moderately Suitability Area		Highly Suitability Area		Total Suitability Area	
	Area (10^4^ km^2^)	Range Expansion (%)	Area (10^4^ km^2^)	Range Expansion (%)	Area (10^4^ km^2^)	Range Expansion (%)	Area (10^4^ km^2^)	Range Expansion (%)
Current	226.0	-	54.1	-	48.3	-	328.4	-
2050s SSP1-2.6	466.5	106.41%	116.8	115.97%	67.1	38.90%	650.4	98.74%
2050s SSP3-7.0	400.0	76.98%	89.4	65.27%	60.2	24.72%	549.6	67.80%
2050s SSP5-8.5	393.5	74.12%	91.4	69.03%	53.4	10.57%	538.4	64.37%
2090s SSP1-2.6	333.6	47.59%	84.9	57.01%	55.1	14.04%	473.6	44.53%
2090s SSP3-7.0	554.3	145.25%	133.3	146.40%	58.0	19.99%	745.5	127.91%
2090s SSP5-8.5	457.5	102.44%	102.1	88.80%	53.9	11.63%	613.6	87.42%

**Table 4:** Spatial changes in the suitable habitat of *C. duclouxiana* under different climate scenarios.

Climate Scenarios	Total Suitability Area (10^4^ km^2^)	Contraction Area (10^4^ km^2^)	Contraction Rate (%)	Unchanged Area (10^4^ km^2^)	Unchanged Rate (%)	Expansion Area (10^4^ km^2^)	Expansion Rate (%)
Current	328.4	-	-	-	-	-	-
2050s SSP1-2.6	650.4	0.0	0.0%	328.4	50.5%	322.1	49.5%
2050s SSP3-7.0	549.6	5.2	1.0%	323.1	58.8%	226.5	41.2%
2050s SSP5-8.5	538.4	0.6	0.1%	327.8	60.9%	210.6	39.1%
2090s SSP1-2.6	473.6	1.2	0.3%	327.2	69.1%	146.4	30.9%
2090s SSP3-7.0	745.5	0.0	0.0%	328.3	44.0%	417.2	56.0%
2090s SSP5-8.5	613.6	6.4	1.0%	321.9	52.5%	291.6	47.5%

## Data Availability

The data are included in the article. For the data provided in this study, see Section 2.1 and Section 2.2 in the text. Further inquiries can be directed to the corresponding author(s).
